# Evolution of a family of metazoan active-site-serine enzymes from penicillin-binding proteins: a novel facet of the bacterial legacy

**DOI:** 10.1186/1471-2148-8-26

**Published:** 2008-01-28

**Authors:** Nina Peitsaro, Zydrune Polianskyte, Jarno Tuimala, Isabella Pörn-Ares, Julius Liobikas, Oliver Speer, Dan Lindholm, James Thompson, Ove Eriksson

**Affiliations:** 1 Research Program of Molecular Neurology, Biomedicum Helsinki, P.O. Box 63, FIN-00014 University of Helsinki, Finland; 2 Helsinki Biophysics and Biomembrane Group, Institute of Biomedicine, P.O. Box 63, FIN-00014 University of Helsinki, Finland; 3 CSC-Scientific Computing Ltd, P.O. Box 405, FIN-02101 Espoo, Finland; 4 Minerva Medical Research Institute, Biomedicum Helsinki, FIN-00290 Helsinki, Finland; 5 Androgen Receptor Laboratory, Institute of Biomedicine, P.O. Box 63, FIN-00014 University of Helsinki, Finland

## Abstract

**Background:**

Bacterial penicillin-binding proteins and β-lactamases (PBP-βLs) constitute a large family of serine proteases that perform essential functions in the synthesis and maintenance of peptidoglycan. Intriguingly, genes encoding PBP-βL homologs occur in many metazoan genomes including humans. The emerging role of LACTB, a mammalian mitochondrial PBP-βL homolog, in metabolic signaling prompted us to investigate the evolutionary history of metazoan PBP-βL proteins.

**Results:**

Metazoan PBP-βL homologs including LACTB share unique structural features with bacterial class B low molecular weight penicillin-binding proteins. The amino acid residues necessary for enzymatic activity in bacterial PBP-βL proteins, including the catalytic serine residue, are conserved in all metazoan homologs. Phylogenetic analysis indicated that metazoan PBP-βL homologs comprise four alloparalogus protein lineages that derive from α-proteobacteria.

**Conclusion:**

While most components of the peptidoglycan synthesis machinery were dumped by early eukaryotes, a few PBP-βL proteins were conserved and are found in metazoans including humans. Metazoan PBP-βL homologs are active-site-serine enzymes that probably have distinct functions in the metabolic circuitry. We hypothesize that PBP-βL proteins in the early eukaryotic cell enabled the degradation of peptidoglycan from ingested bacteria, thereby maximizing the yield of nutrients and streamlining the cell for effective phagocytotic feeding.

## Background

Penicillin-binding proteins and β-lactamases (PBP-βLs) are serine proteases that are distinguished by a catalytic -SXXK-motif (X is any amino acid) [1-5]. Due to their vital role in bacterial biology, PBP-βLs are of importance both medically and economically. Penicillin-binding proteins synthesize and maintain peptidoglycan, the major cell wall component in most bacteria. Penicillin-binding proteins are inhibited by β-lactam antibiotics such as penicillins and cephalosporins which prevent peptidoglycan synthesis and therefore bacterial proliferation. As a defense mechanism against β-lactam antibiotics, some bacteria produce β-lactamases which hydrolyze the antibiotics into biologically inactive metabolites. Phylogenetic analyses show that β-lactamases have evolved from penicillin-binding proteins on at least three occasions indicating a recurrent need to protect/maintain the peptidoglycan synthesis machinery [3,6-8]. Many metazoan organisms including humans harbor proteins that share sequence similarity to PBP-βLs [9]. The genes for metazoan PBP-βL homologs most likely derive from bacteria and may have been acquired by either horizontal or endosymbiotic gene transfer. However, the almost universal lack of peptidoglycan synthesis in eukaryotes raises the questions of (i) what immediate benefit(s) PBP-βL proteins conferred to the recipient cell, and (ii) what biochemical properties the PBP-βL proteins were later endowed with, that lead to their integration in the protein repertoire of higher metazoan species.

Based on amino acid sequence, 3-dimensional structure, and domain organization, bacterial PBP-βLs can be categorized into low molecular weight penicillin-binding proteins classes A to C, high molecular weight penicillin-binding proteins classes A to C, and β-lactamases classes A, C, and D [2-5]. The structure and catalytic mechanism of PBP-βLs have been extensively studied [1-6,10]. All PBP-βLs share three conserved active site motifs which contribute to the formation of the catalytic cavity [1-5]. The -SXXK-motif contains the catalytic serine residue which undergoes acylation and deacylation cycles. The -[SY]X[NT]-motif harbors side chains that point into the active site cleft and participate in the catalytic process. The -[KH][ST]G-motif is located in a β-sheet and participates in substrate docking through antiparallel backbone hydrogen bonding. The arrangement of the three active site motifs along the amino acid sequence is distinctive for each PBP-βL class [2-5]. The size of the PBP-βL domain varies from about 200 amino acids in class D β-lactamases to over 400 amino acids in class C low molecular weight penicillin-binding proteins, indicating that the PBP-βL domain has undergone extensive diversification through modification of local structural elements [1-5].

LACTB is a mammalian protein comprised of a mitochondrial import sequence and a domain sharing sequence similarity to PBP-βLs (human LACTB, [Swiss-Prot:P83111]). This domain is 450 amino acids long and the three PBP-βL active site motifs (-SISK-, -YST-, and -HTG-) have been identified through sequence comparisons with bacterial PBP-βLs [9,11]. LACTB has been detected in several mitochondrial proteome survey studies suggesting that LACTB is a ubiquitous protein in mammalian mitochondria [12-15]. LACTB is subjected to regulation at transcriptional and posttranslational level. In skeletal muscle, LACTB expression is rapidly increased by insulin [16] implying a role in anabolic processes. In liver, lysine acetylation of LACTB occurs during starvation [17] suggesting that LACTB, like several key enzyme in metabolism, is regulated by the highly conserved acetyltransferase/deacetylase pathway [18]. The catalytic serine residue located in the -SISK-motif is phosphorylated under normal conditions suggesting that LACTB is activated by a specific phosphoprotein phosphatase [19]. However, the enzymatic substrates and physiological function of LACTB remains unknown.

Here we have investigated the evolutionary history of LACTB and other metazoan PBP-βLs homologs, which we herein refer to as the LACTB family. A combined phylogenetic and structural analysis demonstrated that the LACTB family has evolved from class B low molecular weight penicillin-binding proteins (LPBP-B). The presence of the conserved catalytic serine residue in all LACTB family proteins suggest that they have peptidase or esterase activity.

## Results and discussion

### LACTB shares conserved active site signature motifs with bacterial PBP-βL proteins

**Figure 1:**
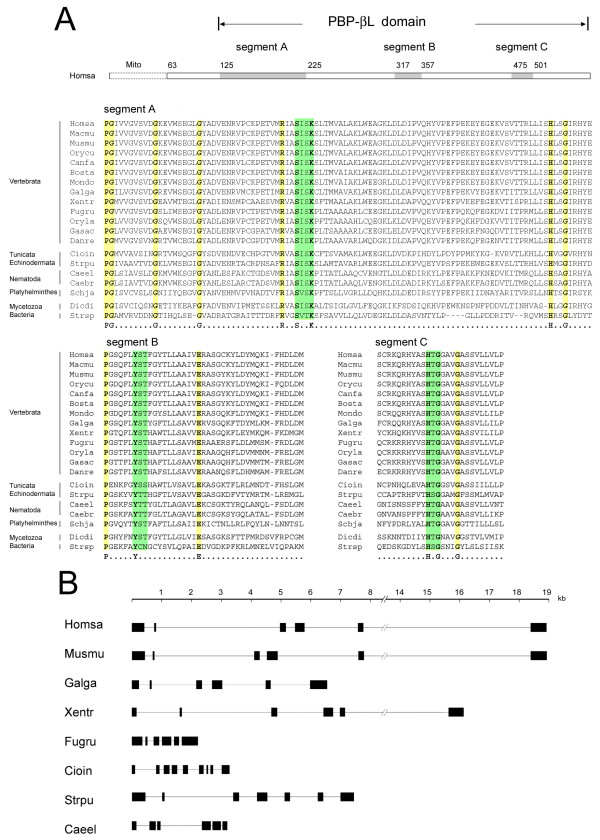
Amino acid alignment of catalytic site motifs and gene architecture of LACTB orthologs. (*A*) Schematic organization and alignment of the three PBP-βLs catalytic site motifs (highlighted in green) and their flanking regions in LACTB orthologs. The corresponding motifs in the *Streptomyces sp. *strain R61 D-alanyl-D-alanine carboxypeptidase [Swiss-Prot:P15555] are included. Abbreviations for species names, in order: Homsa, *Homo sapiens *(Swiss-Prot:P83111); Macmu, *Macaca mulatta *(Ensembl:ENSMMUP00000004719); Musmu, *Mus musculus *(Swiss-Prot:Q9EP89); Orycu, *Oryctolagus cuniculus *(Ensembl:ENSOCUP00000008608); Canfa, *Canis familiaris *(RefSeq:XP_544713); Bosta, *Bos taurus *(Swiss-Prot:P83095); Mondo, *Monodelphis domestica *(Ensembl:ENSMODP00000013893); Galga,*Gallus gallus *(Swiss-Prot:Q5ZK12); Xentr, *Xenopus tropicalis *(Ensembl:ENSXETG00000009720); Fugru, *Fugu rubipes *(Ensembl:SINFRUP00000138119); Oryla, *Oryzia latipes *(Ensembl:ENSORLP00000009787); Gasac, *Gasterosteus aculeatus *(Ensembl:ENSGACP00000014046); Danre, *Danio rerio *(RefSeq:NP_001018429); Strpu, *Strongylocentrotus purpuratus *(RefSeq:XP_789736); Cioin, *Ciona intestinalis *(Ensembl:ENSCING00000006798); Schja, *Schistosoma japonicum *(GenPept:AAX27853, AAX25200); Caeel, *Caenorhabditis elegans *(RefSeq:NP_001041033); Caebr, *Caenorhabiditis briggsae *(GenPept:CAE74593); Dicdi, *Dictyostelium discoideum *(Swiss-Prot:Q55CN2); Stesp, *Streptomyces sp *strain R61. The mitochondrial import sequence (*Mito*) is indicated. Amino acid conserved in all taxa are highlighted in yellow. (*B*) Organization of exons and introns in LACTB genes of representative metazoan taxa.

We performed extensive database searches for proteins sharing sequence similarity with human LACTB. Metazoan proteins containing the three active site motifs -SXXK-, -[SY]X[NT]-, and -[KH][ST]G-shared by all PBP-βLs [1-6], were classified as LACTB family proteins (Table 1). LACTB orthologs were identified using the reciprocal best-hit approach [20,21]. We found that all completed vertebrate genomes harbor a LACTB ortholog. We also identified LACTB orthologs in *Ciona intestinalis*, *Strogylocentrotus purpuratus*, *Caenorhabditis elegans*, *Caenorhabditis briggsae*, *Schistosoma japonicum*, and *Dictyostelium discoideum*. An amino acid alignment of the active site motifs and their flanking regions is shown (Figure 1, panel *A*). For comparison we included the corresponding amino acid segments from the D-alanyl-D-alanine carboxypeptidase [Swiss-Prot:P15555] of the actinobacterium *Streptomyces sp. *strain R61, which is perhaps the most extensively studied PBP-βL family protein [22,23]. The alignment shows that the active site motifs and the catalytic serine residue are conserved in all taxa from bacteria to human suggesting that LACTB is an active-site-serine enzyme. In addition, several amino acids flanking the active site motifs are conserved from bacteria to human further suggesting that LACTB and the bacterial PBP-βL proteins share common features in the secondary and tertiary structure. Metazoan LACTB orthologs harbor a 50–100 amino acid long N-terminal region with no sequence similarity to the *Streptomyces *D-alanyl-D-alanine carboxypeptidase or to any other bacterial PBP-βL protein. The N-terminal region starts with a predicted mitochondrial import sequence (Figure 1, panel *A*). Comparison of the gene architecture of the metazoan LACTB orthologs (Figure 1, panel *B*) revealed extensive similarities of the exon-intron organization supporting that these genes were vertically inherited from a common ancestor [24].

**Table 1:** Accession Numbers and Proposed Classification of Proteins Used in This Study

Species	Accession number	Database	Proposed classification	Reference
**EUKARYOTA**				
**Vertebrata**				
*Bos taurus*	P83095	Swiss-Prot	LACTB	[12]
*Bos taurus*	XP_601949	RefSeq	LACTB-like protein 2	
*Canis familiaris*	XP_544713	RefSeq	LACTB	[47]
*Danio rerio*	NP_001018429	RefSeq	LACTB	
*Fugu rubipes*	SINFRUP00000138119	Ensembl	LACTB	
*Gallus gallus*	Q5ZK12	Swiss-Prot	LACTB	[48]
*Gallus gallus*	XP_001232283	RefSeq	LACTB-like protein 2	[48]
*Gasterosteus aculeatus*	ENSGACP00000014046	Ensembl	LACTB	
*Homo sapiens*	P83111	Swiss-Prot	LACTB	[9]
*Homo sapiens*	XP_934300	RefSeq	LACTB-like protein 2	
*Macaca mulatta*	ENSMMUP00000004719	Ensembl	LACTB	
*Monodephis domestica*	ENSMODP00000013893	Ensembl	LACTB	
*Mus musculus*	Q9EP89	Swiss-Prot	LACTB	[11,13]
*Mus musculus*	XP_205950	RefSeq	LACTB-like protein 2	
*Oryctolagus cuniculus*	ENSOCUP00000008608	Ensembl	LACTB	
*Oryzias latipes*	ENSORLP00000009787	Ensembl	LACTB	
*Tetrodon nigroviridis*	CAG03509	GenPept	LACTB-like protein 2	[49]
*Tetrodon nigroviridis*	GSTENP00015949001	Ensembl	LACTB	[49]
*Xenopus tropicalis*	ENSXETG00000009720	Ensembl	LACTB	
**Tunicata**				
*Ciona intestinalis*	ENSCING00000006798	Ensembl	LACTB	[50]
**Echinodermata**				
*Strongylocent. purpuratus*	XP_781179	RefSeq	Esterase-like	
*Strongylocent. purpuratus*	XP_781241	RefSeq	Esterase-like	
*Strongylocent. purpuratus*	XP_789736	RefSeq	LACTB	
**Nematoda**				
*Caenorhabditis briggsae*	CAE57335	GenPept	LACTB-like protein 1	[51]
*Caenorhabditis briggsae*	CAE57450	GenPept	Esterase-like	[51]
*Caenorhabditis briggsae*	CAE59629	GenPept	Esterase-like	[51]
*Caenorhabditis briggsae*	CAE67822	GenPept	Esterase-like	[51]
*Caenorhabditis briggsae*	CAE73201	GenPept	Esterase-like	[51]
*Caenorhabditis briggsae*	CAE74593	GenPept	LACTB	[51]
*Caenorhabditis briggsae*	CAE75151	GenPept	LACTB-like protein 1	[51]
*Caenorhabditis elegans*	NP_001041033	RefSeq	LACTB	[52]
*Caenorhabditis elegans*	NP_495790	RefSeq	Esterase-like	[52]
*Caenorhabditis elegans*	NP_496176	RefSeq	Esterase-like	[52]
*Caenorhabditis elegans*	NP_496299	RefSeq	Esterase-like	[52]
*Caenorhabditis elegans*	NP_505821	RefSeq	LACTB-like protein 1	[52]
*Caenorhabditis elegans*	NP_509221	RefSeq	Esterase-like	[52]
*Caenorhabditis elegans*	NP_509969	RefSeq	LACTB-like protein 1	[52]
**Platyhelminthes**				
*Schistosoma japonicum*	AAX27853, AAX25200	GenPept	LACTB	
**Mycetozoa**				
*Dictyostelium discoideum*	Q55CN2	Swiss-Prot	LACTB	[53]
*Dictyostelium discoideum*	XP_640123	RefSeq	LACTB-like protein 2	[53]
*Dictyostelium discoideum*	XP_640124	RefSeq	LACTB-like protein 2	[53]
*Dictyostelium discoideum*	XP_641364	RefSeq	LACTB-like protein 2	[53]
				
**BACTERIA**				
**α-proteobacteria**				
*Bradyrhizobium japonicum*	NP_770552	RefSeq	LPBP-B^a^	
*Hyphomonas neptunium*	YP_759849	RefSeq	LPBP-B^a^	
*Maricaulis maris*	YP_755834	RefSeq	LPBP-B^a^	
*Mesorhizobium loti*	NP_107127	RefSeq	LPBP-B^a^	
*Oceanicaulis alexandrii*	YP_755834	RefSeq	LPBP-B^a^	
*Sphingopyxis alaskensis*	YP_618034	RefSeq	LPBP-B^a^	
**β-proteobacteria**				
*Burkholderia gladioli*	Q9KX40	Swiss-Prot	LPBP-B^a^	
**Bacillae**				
*Bacillus cereus*	ZP_00239307	RefSeq	LPBP-B^a^	
**Actinobacteria**				
*Streptomyces sp. *strain R61	P15555	Swiss-Prot		[22,23]
*Salinispora tropica*	ZP_01429613	RefSeq	LPBP-B^a^	

NOTE.- ^a^Classified according to the scheme of Massova and Mobashery [3].

### Amino acid sequence and structural features indicate that LACTB derives from class B low molecular weight penicillin-binding proteins

**Figure 2:**
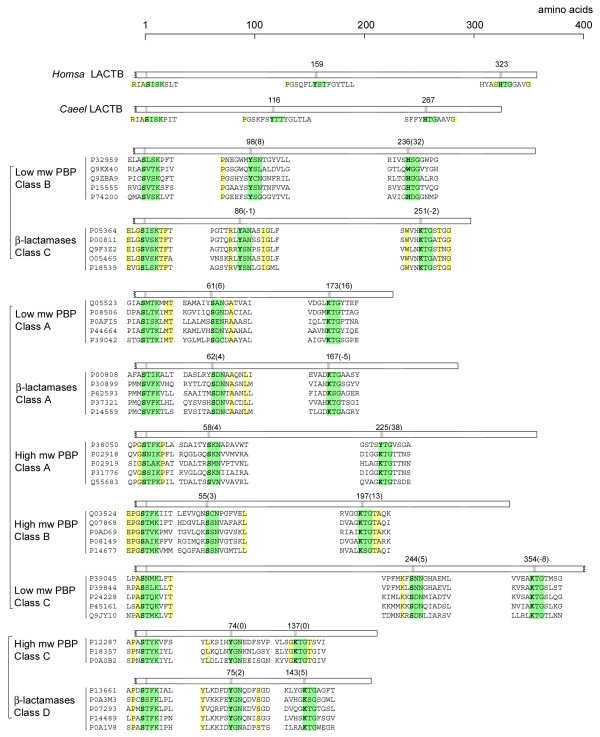
Schematic representation of the organization of the three catalytic site motifs in LACTB and the different PBP-βL classes. A set of founding members of each PBP-βL class (Additional file 1), classified according to Ghuysen 1997, and Massova and Mobashery 1998 [3,4], were used to calculate the median inter-motif distances in number of amino acid residues. Accession numbers refer to the Swiss-Prot database. The catalytic site motifs are highlighted in green and invariant amino acids are higlighted in yellow. Inter-motif distances were measured from the serine in the -SXXK-motif to the serine/lysine and lysine/histidine of the second and third catalytic site motif, respectively. Numbers within brackets is the largest difference from the median value within each class. PBP-βL classes forming separate clades [3] are marked with square brackets. Abbreviations: PBP, penicillin-binding protein.

Phylogenetic and structural analyses show that the bacterial PBP-βL family is monophyletic and has diversified by local structural changes and domain fusions [1-8]. The bacterial PBP-βL family can be divided into seven distinct and highly divergent classes that share little or no inter-class sequence identity except for the three common catalytic site motifs [2-8]. Proteins from the different PBP-βL classes are distinguished by the specific amino acids in the active site motifs and the number of amino acid residues between the three motifs [2-8]. The arrangement of the active site motifs along the amino acid sequence for a set of founding members of each bacterial PBP-βL class is shown (Figure 2, Additional file 1). Comparing the arrangement of the active site motifs and their specific amino acids in human and *C. elegans *LACTB with the founding members of each of the bacterial PBP-βL classes revealed that LACTB is related to the LPBP-B proteins. Sequence comparison of the PBP-βL domain of LACTB with the LPBP-B proteins indicated a moderate degree of sequence identity (16–27%). Conversely, there was little or no inter-motif similarity between LACTB and the proteins belonging to the other bacterial PBP-βL classes. Based on comparison with the founding members of the PBP-βL classes, LACTB can thus be classified as a LPBP-B protein. To test the global validity of this classification we investigated if all bacterial PBP-βL proteins sharing sequence similarity to LACTB are LBPB-B proteins. Searches in the NCBI nr database using human LACTB as the seed sequence yielded more than five hundred bacterial proteins. We divided these proteins into five categories based on E-value (10^-5^–10^-10^, 10^-10^–10^-20^, 10^-20^–10^-30^, 10^-30^–10^-40^, 10^-40 ^and lower). Ten proteins randomly chosen from each category were aligned with the founding members of the LBPB-B group. All the sampled bacterial proteins, irrespective of E-value, had a characteristic LPBP-B type motif organization, shared 25–95% sequence identity with the founding members of the LPBP-B class and had little or no sequence similarity with other PBP-βL classes. This result suggests that LACTB together with the LPBP-B proteins form a distinct class sharing a common ancestry.

At present, 3-dimensional structures are available for three LPBP-B class proteins: the *Streptomyces *D-alanyl-D-alanine carboxypeptidase [22,23]; a D-alanyl-D-alanine aminopeptidase from *Ochrobactrum anthropii *[Swiss-Prot:Q9ZBA9] [25]; and a transesterase from *Burkholderia gladioli *[Swiss-Prot:Q9KX40] [26]. These three proteins representing different LPBP-B subclasses share an almost identical polypeptide fold consisting of an α/β region and an all-helical region, with the catalytic site located between them. Comparison of the amino acid sequences with the 3-dimensional structures of these LPBP-B proteins revealed several conserved amino acids in structurally critical elements of the polypeptide fold (Additional file 2). Amino acid alignments of LACTB with these LPBP-B proteins showed a high degree of amino acid conservation especially of amino acids in positions corresponding to these structural elements (Additional file 2). Evidently, local structure imposes particularly stringent constraints on substitutions in these positions promoting their conservation through long evolutionary distances. These findings strongly suggest that LACTB and the LPBP-B proteins share a similar polypeptide fold.

### The LACTB family comprises four paralogus lineages

Having identified a set of LACTB orthologs we sought to determine the evolutionary relationship with other LACTB family proteins (Table 1). Amino acid sequence comparisons revealed that all LACTB family proteins have a LPBP-B-like motif arrangement and, moreover, share a 17–40% inter-motif sequence identity to human LACTB (Additional file 3). This finding suggests that the LACTB family descended from a common bacterial ancestor protein and then diversified in various metazoan lineages. The most extensive diversification of the LACTB family occurred in nematodes which have eight paralogs, whilst vertebrates harbor two paralogs. In contrast, all insects sequenced to date appear to lack LACTB proteins. To gain further insight into the evolutionary events shaping the LACTB family we performed a phylogenetic analysis.

**Figure 3:**
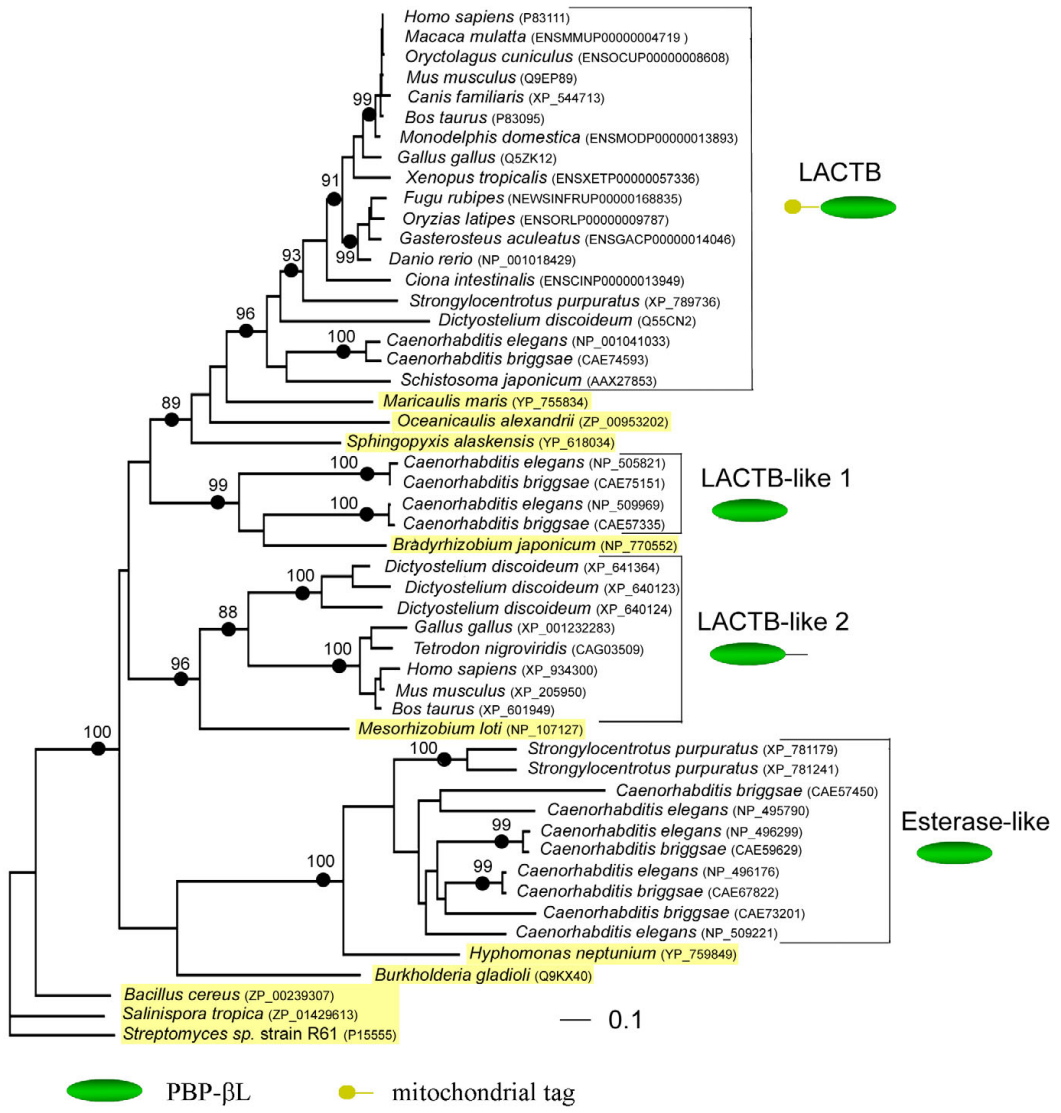
Inferred phylogenetic tree of the PBP-βL domain of LACTB family proteins. An alignment encompassing 266 amino acids was analyzed by maximum likelihood as described in the Materials and Methods section. The log likelihood was -20487.28 and the Γ distribution shape parameter shape parameter used was 2.670. Bootstrap values for nodes supported by more than 85 replicates of 100 are shown. Bacterial proteins are highlighted in yellow.

The PBP-βL domain of thirty-seven metazoan LACTB family proteins, four *Dictyostelium *LACTB homologs, and ten bacterial LPBP-B proteins were aligned and analyzed by maximum likelihood as described in Methods. The *Streptomyces *D-alanyl-D-alanine carboxypeptidase and two related LPBP-B proteins from *Salinispora tropica *and *Bacillus cereus *(E < 10^-50 ^against *Streptomyces*) were included as an outgroup (Table 1). The inferred best tree, shown in Figure 3, indicated that the LACTB family is composed of four groups. We have labeled the groups: (i) LACTB orthologs; (ii) LACTB-like 1; (iii) LACTB-like 2; and (iv) esterase-like proteins (Figure 3). These groups were supported by a bootstrap analysis of one hundred replicates. That the bacterial LPBP-B proteins were intercalated with the different LACTB groups, instead of forming a sister group common to all LACTB groups, suggests that the LACTB family is composed of four distinct alloparalogus protein lineages.

### The LACTB family derives from α-proteobacterial ancestor proteins

LACTB orthologs clustered together with LPBP-B proteins from the free-living α-proteobacteria *Maricaulis maris*, *Oceanicaulis alexandrii*, and *Sphingopyxis alaskensis*. This finding suggests that the progenitor of the LACTB orthologs was acquired from an early α-proteobacterium. Interestingly, the evolutionary scenario appears to be similar for the other LACTB groups. Esterase-like proteins cluster with a putative esterase from *Hyphomonas neptunium*, the LACTB-like group 1 and 2 proteins cluster with putative peptidases from *Bradyrhizobium japonicum *and *Mesorhizobium loti*, respectively. These findings suggest that the progenitors for the LACTB family were acquired simultaneously from an early α-proteobacterium harboring a set of four LPBP-B genes encoding structurally closely related proteins with different biochemical functions. That the LACTB groups cluster with proteins from different α-proteobacterial lineages, may be explained by lineage-specific loss of LPBP-B genes resulting from the extensive genome reductions that has occurred throughout the evolution of α-proteobacteria [27].

While LACTB orthologs have a relatively broad taxon distribution, proteins from the other three lineages are restricted to fewer taxa suggesting that widespread loss of these genes have occurred throughout the eukaryotic evolution. LACTB-like proteins 1 are found only in nematodes suggesting gene loss in all other lineages. In contrast, LACTB-like proteins 2 are abundant in *Dictyostelium *and comprise several vertebrate proteins, but do not occur in nematodes. The esterase-like proteins comprise five paralogs in nematodes and two in echinoderms, but appears to be missing from all vertebrates sequenced to date.

## Conclusion

The PBP-βL family encompasses a large number of highly diversified proteins in bacteria and eukaryotes. While the role of bacterial PBP-βL proteins in peptidoglycan synthesis has been extensively studied, little is known about the function of metazoan PBP-βL family proteins. However, recent findings show that the mammalian mitochondrial PBP-βL homolog LACTB is involved in metabolic signaling [16,17,19]. Therefore, clarifying the function of metazoan PBP-βL homologs may reveal novel aspects about the evolution of metazoan energy metabolism and elucidate unknown mechanisms of metabolic regulation.

Our phylogenetic analysis indicated that the LACTB family is divided into four lineages deriving from four separate bacterial LPBP-B subclass genes. These genes were most likely acquired simultaneously from α-proteobacteria by endosymbiotic gene transfer, although other scenarios such as multiple horizontal gene transfers from α-proteobacteria to early organisms of the opistokont lineage can not be completely ruled out. The evolutionary history of the LACTB family is dominated by gene losses resulting in an uneven distribution of LACTB family proteins in metazoan taxa and no extant organism sequenced to date harbor proteins from all four lineages. Extensive diversification of LACTB family proteins appear to have occurred only in Nematodes. Since metazoan organisms lack peptidoglycan the widespread gene loss characterizing the history of the LACTB family may be readily explained by lack of enzymatic substrates. However, this raises the question as to what mechanisms have promoted the conservation of PBP-βL homologs in some eukaryotic lineages.

We speculate that phagocytotic feeding was the primary selection force acting to conserve genes for LPBP-B proteins in early eukaryotes. An early eukaryote organism possessing molecular machineries for both phagocytosis and effective peptidoglycan digestion would harness great benefit in an environment with numerous bacteria. It is thus possible that this hypothetical early eukaryote was endowed not only with the progenitors of the LACTB family proteins but was equipped with a larger complement of PBP-βL proteins and other peptidoglycan-degrading enzymes allowing an efficient and complete hydrolysis of ingested peptidoglycan. Following the diversification of eukaryotes, lineages in which phagocytotic feeding was deselected had no need for peptidoglycan-degrading enzymes and they were consequently lost from the genomes. On the contrary, eukaryote lineages leading to *Dictyostelium *and *Caenorhabditi*s, which feed on bacteria, retained the genes for peptidoglycan-digesting enzymes and allowed them to undergo multiple duplications. This scenario implies that LACTB family proteins may also occur in some phagotropic protozoans.

LACTB orthologs are conserved in many metazoan species including all vertebrates currently sequenced. The energy metabolism of vertebrates is intimately linked to the metabolism of the large number of microbes that colonize the intestinal channel. That changes in the relative abundance of intestinal microbes from different taxa directly affects the lipid and carbohydrate metabolism of the host ([28] and references therein) demonstrates the existence of complex gut-microbe-to-host signaling mechanisms relayed by specific microbial metabolites. We hypothesize that LACTB is involved in the metabolism and/or sensing of some product(s) deriving from commensal bacteria. Further metagenomic, cell biological, and biochemical studies will be required to elucidate the function of PBP-βL homologs in metazoan organisms.

## Methods

Genomic DNA, expressed sequence tags, and protein databases were searched for nucleotide and amino acid sequences using BLAST 15/10/2006–15/01/2007. The following websites were used; Dictybase [29], DOE Joint Genome Institute [30], J. Craig Venter Institute [31], H-invitational database [32], Human Genome Sequencing Center [33], LumbriBASE [34], National Center for Biotechology Information [35], Washington University School of Medicine Genome Sequencing Center [36], Wellcome Trust Sanger Institute [37], and Wormbase [38].

Orthologs to human LACTB were identified using a modified reciprocal best-hit approach as described [20,21]. To identify bacterial orthologs each LACTB family protein was used to search the NCBI nr database. Retrieved bacterial proteins were evaluated with the reciprocal best-hit test. Expectancy values (E-value) refer to searches in the NCBI nr sequence database. Binary alignments were made using Dotlet 1.5 [39] and LALIGN [40]. Multiple alignments of amino acid sequences were made using ClustalW and adjusted manually. Amino acid alignments are available upon request.

Phylogenetic maximum likelihood analysis of amino acid alignments was performed in software programs RAxML-VI [41] and proml from the PHYLIP package version 3.66 [42]. The invariant sites were removed from the alignment and the shape parameter of the Γ distribution was estimated using ProtTest [43]. Four Γ distribution classes with the JTT model were used in both programs. Trees were initially inferred in RAxML using the normal hill-climbing search with 100 random addition sequences. The resulting trees were further evaluated in proml, and the one giving the highest likelihood value is presented. The robustness of inferred trees was assessed by nonparametric bootstrapping using programs seqboot, proml, and consense from the PHYLIP package. One hundred replicates was used in the bootstrapping analysis. Like the majority of eukaryotic operational genes with bacterial homologs the LACTB family branched with homologs from Gram negative bacteria while homologs from Gram positive bacteria formed an outgroup. Therefore the *Streptomyces sp. *D-alanyl-D-alanine carboxypeptidase [Swiss-Prot:P15555] was used to root the inferred tree.

The probability of mitochondrial import was estimated using Mitoprot mitochondrial targeting sequence prediction [44,45]. The Prosite [46] nomenclature for amino acid patterns was used.

## Abbreviations

LPBP-B, low molecular weight penicillin-binding protein class B; PBP-βL, penicillin-binding protein and β-lactamase.

## Authors' contributions

NP and ZP contributed equally to this study. All authors read and approved the manuscript.

## Supplementary Material

Additional file 1 Accession Numbers and Classification of a Set of Founding Members of the PBP-βL classes. Contains a list of founding members of the different PBP-βL classes including additional references. Click here for file

Additional file 2 Conserved Structural Elements in LPBP-Bs and LACTB. Contains a list of amino acids in conserved motifs found in LPBP-B proteins and LACTB. Click here for file

Additional file 3 Conserved PBP-βL signature motifs in LACTB family proteins. Multiple amino acid alignment of PBP-βL signature motif-containing segments from LACTB family proteins. Click here for file
