# Supplementary material for: Evolution of a family of metazoan active-site-serine enzymes from penicillin-binding proteins: a novel facet of the bacterial legacy
Source: BMC Evol Biol. 2008 Jan 28;8:26. doi: 10.1186/1471-2148-8-26 (PMC2266909; doi:10.1186/1471-2148-8-26)
Supplement: Additional file 1 — Accession Numbers and Classification of a Set of Founding Members of the PBP-βL classes. Contains a list of founding members of the different PBP-βL classes including additional references. [file 1471-2148-8-26-S1.doc]

**Additional file 1**

**Accession Numbers and Classification of a Set of Founding Members of the PBP-βL classes**

**________________________________________________________________________**

**PBP-βL class and source Swiss-Prot no. References**

____________________________________________________________________________________________________________

**LPBP-A**

*Bacillus stearotermophilus* Q05523 [54]

*Escherichia coli* P08506 [55]

*Escherichia coli* P0AFI5 [56]

*Haemophilus influenzae* P44664 [3]

*Streptomyces* K15 P39042 [57]

**LPBP-B**

*Bacillus subtilis* P32959 [58]

*Burkholderia gladioli* Q9KX40 [26]

*Ochrobactrum antropi* Q9ZBA9 [25]

*Streptomyces* sp*.* strain R61 P15555 [22,23]

*Synechocystis* sp. strain PCC6803 P74200 [3]

**LPBP-C**

*Actinomadura* sp. R39 P39045 [59]

*Bacillus subtilis* P39844 [3]

*Escherichia coli* P24228 [60]

*Haemophilus influenzae* P45161 [3]

*Neisseria meningitidis* Q9JY10 [3]

**HPBP-A**

*Bacillus subtilis* P38050 [61]

*Escherichia coli* P02918 [62]

*Escherichia coli* P02919 [63]

*Haemophilus influenzae* P31776 [64]

*Synechocystis* sp. strain PCC6803 Q55683 [3]

**HPBP-B**

*Bacillus subtilis* Q03524 [3]

*Bacillus subtilis* Q07868 [65]

*Escherichia coli* P0AD69 [3]

*Neisseria gonorrhoeae* P08149 [66]

*Streptococcus pneumoniae* P14677 [67]

**HPBP-C**

*Bacillus licheniformis* P12287 [68]

*Staphylococcus aureus* P18357 [69]

*Staphylococcus epidermidis* P0A0B2 [3]

**LacA**

*Bacillus licheniformis* P00808 [70]

*Bacteroides vulgatus* P30899 [71]

*Escherichia coli* P62593 [72]

*Pseudomonas aeruginosa* P37321 [73]

*Streptomyces albus*G P14559 [3]

**LacC**

*Enterobacter cloacae* P05364 [74]

*Escherichia coli* P00811 [75]

*Ochrobactrum antropi* Q9F3Z2 [76]

*Psychrobacter immobilis* O05465 [77]

*Serratia marcescens* P18539 [78]

**LacD**

*Escherichia coli* P13661 [79]

*Klebsiella pneumoniae* P0A3M3 [80]

*Pseudomonas aeruginosa* O07293 [81]

*Pseudomonas aeruginosa* P14489 [82]

*Salmonella typhimurium* P0A1V8 [83]

____________________________________________________________________________________________________________

**Additional references**

1. Despreaux CW, Manning RF: **The *dacA* gene of *Bacillus stearothermophilus* coding for D-alanine carboxypeptidase: cloning, structure and expression in *Escherichia coli* and *Pichia pastoris*.** *Gene 1993,* **131:**35-41.
2. Nicholas RA, Krings S, Tomberg J, Nicola G, Davies C: **Crystal structure of Wild-type Penicillin-binding Protein 5 from *Escherichia coli*. Implications for deacylation of the acyl-enzyme complex.** *J Biol Chem 2003,* **278:**52826-52833.
3. Romeis T, Höltje J-V: **Penicillin-binding protein 7/8 of *Escherichia coli* is a dd-endopeptidase.** *Eur J Biochem 1994,* **224:**597-604.
4. Rhazi N, Charlier P, Dehareng D, Engher D, Vermiere M, Frère J-M, Nguyen-Distèche M, Fonzé E: **Catalytic Mechanism of the *Streptomyces* K15 dd-Transpeptidase/Penicillin-Binding Protein Probed by Site-Directed Mutagenesis and Structural Analysis.** *Biochemistry 2003,* **42:**2895-2906.
5. Popham DL, Setlow P: **Cloning, Nucleotide Sequence, and Regulation of the *Bacillus subtilis pbpE* Operon, Which Codes for Penicillin-Binding Protein 4* and an Apparent Amino Acid Racemase.** *J Bacteriol 1993,* **175:**2917-2925.
6. Sauvage E, Herman R, Petrella S, Duez C, Bouilenne F, Frère J-M, Charlier P: **Crystal structure of the *Actinomadura* R39 dd-peptidase Reveals New Domains in Penicillin-binding Proteins.** *J Biol Chem 2005,* **280:**31249-31256.
7. Kishida H, Unzai S, Roper DI, Lloyd A, Part S-Y, Tame JRH: **Crystal Structure of Penicillin Binding protein 4 (dacB) from *Escherichia coli,* both in the Native Form and Covalently Linked to Various Antibiotics.** *Biochemistry 2006,* **45:**783-792.
8. Popham DL, Setlow P: **Cloning, Nucleotide Sequence, and Regulation of the *Bacillus subtilis pbpE* Gene, Which Codes for a Putative Class A High-Molecular-Weight Penicillin-Binding Protein.** *J Bacteriol 1993,* **175:**4870-4976.
9. Keck W, Glauner B, Schwarz U, Broome-Smith JK, Spratt BG: **Sequences of the Active-Site Peptides of Three of the High-Mr Penicillin-Binding Proteins of Escherichia coli K-12.** *Proc Natl Acad Sci USA 1985,* **82:**1999-2003.
10. Terrak M, Ghosh TK, van Heijenoort J, Van Beeumen J, Lamplias M, Aszodi J, Ayala JA, Ghuysen J-M, Nguyen-Distèche M: **The catalytic, glycosyl transferase and acyl transferase modules of the cell wall peptidoglycan-polymerizing penicillin-binding protein 1b of *Escherichia coli*.** *Mol Microbiol 1999,* **34:**350-364.
11. Sharma UK, Dwarakanath P, Banerjee N, Town C, Balganesh TS: **Expression and Characterization of the *ponA* (ORF I) Gene of *Haemophilus influenzae*: Functional Complementation in a Heterologous.** *System J Bact 1995,* **177:**6745-6750.
12. Yanouri A, Daniel RA, Errington J, Buchanan CE: **Cloning and Sequencing of the Cell Division Gene *pbpB*, Which Encodes Penicillin-Binding Protein 2B in  *Bacillus subtilis*.** *J Bacteriol 1993,* **175:**7604-7616.
13. Spratt BG: **Hybrid penicillin-binding proteins in penicillin-resistant strains of *Neisseria gonorrhoeae*.** *Nature 1988,* **332:**173-176.
14. Dessen A, Mouz N, Gordon E, Hopkins J, Dideberg O: **Crystal Structure of PBP2x from Highly Penicillin-resistant *Streptococcus pneumoniae* Clinical Isolate.** *J Biol Chem 2001,* **276:**45106-45112.
15. Kerff F, Charlier P, Colombo M-L, Sauvage E, Brans A, Frère J-M, Joris B, Fonzé E: **Crystal Structure of the Sensor Domain of the BlaR Penicillin Receptor from *Bacillus licheniformis*.** *Biochemistry 2003,* **42:**12835-12843.
16. Wilke MS, Hills TL, Zhang HZ, Chambers HF, Strynadka NC: **Crystal Structures of the Apo and Penicillin-acylated Forms of the BlaR1 β-Lactam Sensor of *Staphylococcus aureus*.** *J Biol. Chem 2004,* **279:**47278-47287.
17. Fonze E, Vanhove M, Dive G, Sauvage E, Frère J-M, Charlier P: **Crystal Structures of the *Bacillus Licheniformis* BS3 Class A β-Lactamase and of the Acyl-Enzyme Adduct Formed with Cefoxitin.** *Biochemistry 2002,* **41:**1877-1885.
18. Parker AC, Smith CJ: **Genetic and Biochemical Analysis of a Novel Ambler Class A β-Lactamase Responsible for Cefoxitin Resistance in *Bacteroides*  Species.** *Antimicrob. Agents Chemother 1993,* **37:**1028-1036.
19. Maveyraud L, Pratt RF, Samama J-P: **Crystal structure of an acylation transition-state analog of the TEM-1 beta-lactamase. Mechanistic implications for class A beta-lactamases.** *Biochemistry 1998,* **37:**2622-2628.
20. Nordmann P, Naas T: **Sequence analysis of PER-1 extended-spectrum beta-lactamase from *Pseudomonas aeruginosa* and comparison with class A beta-lactamases.**
    *Antimicrob Agents Chemother 1994,* **38:**104-114.
21. Lobovsky E, Moews PC, Liu H, Zhao H, Frere J-M, Knox JR: **Evolution of an enzyme activity: Crystallographic structure at 2-Å resolution of cephalosporinase from the *ampC* gene of *Enterobacter cloacae* P99 and comparison with a class A penicillinase.** *Proc Natl Acad Sci USA 1992,* **90:**11257-11261.
22. Tondi D, Powers RA, Caselli E, Negri MC, Blazquez J, Costi MP, Shoichet BK: **Structure-Based Design and in-Parallel Synthesis of Inhibitors of AmpC β-Lactamase.** *Chem Biol 2001,* **8:**593-610.
23. Higgins CS, Avison MB, Jamieson L, Simm AM, Bennett PM, Walsh TR: **Characterization, cloning and sequence analysis of the inducible *Ochrobactrum anthropi* AmpC β-lactamase.** *J Antimicrob Chemother 2001,* **47:**745-754.
24. Feller G, Zekhnini Z, Lamotte-Brasseur J, Gerday C: **Enzymes from cold-adapted microorganisms The class C β-lactamase from the antarctic psychrophile *Psychrobacter immobilis* A5.** *Eur J Biochem 1997,* **244:**186-191.
25. Nomura K, Yoshida T: **Nucleotide sequence of the *Serratia marcescens* SR50 chromosomal ampC beta-lactamase gene.** *FEMS Microbiol Lett 1990,* **58:**295-299.
26. Sun T, Nukaga M, Mayama K, Braswell EH, Knox JR: **Comparison of β-Lactamases of classes A and D: 1.5-Å crystallographic structure of the class D OXA-1 oxacillinase.** *Protein Sci 2003,* **12:**82-91.
27. Sarno R, McGillivary G, Sherratt DJ, Actis LA, Tolmasky ME. **Complete nucleotide sequence of Klebsiella pneumoniae multiresistance plasmid pJHCMW1.**
    *Antimicrob Agents Chemother 2002,* **46:**3422-3427.
28. Philippon LN, Naas T, Bouthors AT, Barakett V, Nordmann P: **OXA-18, a class D Clavulanic Acid-Inhibited Extended-Spectrum β-lactamase from *Pseudomonas aeruginosa*.** *Antimicrob Agents Chemother 1997,* **41:**2188-2195.
29. Maveyraud L, Golemi D, Kotra LP, Tranier S, Vakulenko S, Mobashery S, Samama J-P: **Insights into class D β-lactamases are revealed by the crystal structure of the OXA10 enzyme from *Pseudomonas aeruginosa*.** *Structure 2000,* **8:**1289-1298.
30. Dale JW, Godwin D, Mossakowska D, Stephenson P, Wall S: **Sequence of the OXA2 beta-lactamase: comparison with other penicillin-reactive enzymes.** *FEBS Lett 1985,* **191:**39-44.
31. Joris B, Ledent P, Dideberg O, Fonzé E, LaMotte-Brasseur J, Kelly JA, Ghuysen JM, Frère JM: **Comparison of the Sequences of Class A β-Lactamases and of the secondary Structure Elements of Penicillin-Recognizing Proteins.** *Antimicrob Agents Chemother 1991,* **35:**2294-2301.
