# Supplementary material for: Evolution of a family of metazoan active-site-serine enzymes from penicillin-binding proteins: a novel facet of the bacterial legacy
Source: BMC Evol Biol. 2008 Jan 28;8:26. doi: 10.1186/1471-2148-8-26 (PMC2266909; doi:10.1186/1471-2148-8-26)
Supplement: Additional file 2 — Conserved Structural Elements in LPBP-Bs and LACTB. Contains a list of amino acids in conserved motifs found in LPBP-B proteins and LACTB. [file 1471-2148-8-26-S2.doc]

**Additional file 2**

**Conserved Structural Elements in LPBP-Bs and LACTB**

**________________________________________________________________________**

**Structural element^a^ Source and accession code (Swiss-Prot and PDB)**

**________________________________________________**

*Streptomyces Ochrobactrum Burkholderia Homo sapiens*

**P15555 Q9ZBA9 Q9KX40 P83111^b^**

**1CEF 1EI5 1CI8**

_____________________________________________________________________________________________________________________________________

Curve on beta strand B1 P24-G25 P23-G24 V36-G37 P125-G126

Hairpin loop between beta strands B1 and B2 G34 G33 G46 G135

Curve on beta strand B2 G42 G42 G55 G144

Catalytic site motif I S62-K65 S62-K65 S75-K78 S164-K167

Loop after helix H2 G79 G77 G92 G181

Loop after helix H3b H118-G111 N106-G109 H127-G130 H216-G219

Surface loop P153-G154 P147-G148 P175-G176 P317-G318

Catalytic site motif II Y159-N161 Y153-N155 Y181-L183 Y323-T325

Loop between helices H5 and H6 G176 G170 G197 G340

First turn of helix H8 D246 D234 D283 D406

Hairpin loop between beta strands B2e and B2f G283 G273 L327 A463

Catalytic site motif III H298-G300 H287-G299 W348-G350 H485-G487

Curve on beta strand B5 N324 N313 N371 N524

____________________________________________________________________________________________________________

Note.-^a^The classification of secondary structure elements was made according to Lobovsky et al. [74] and Joris et al. [84].

^b^For LACTB the positions of the structural elements were inferred by amino acid sequence comparison.
