# Supplementary figures and images for: Evolution of a family of metazoan active-site-serine enzymes from penicillin-binding proteins: a novel facet of the bacterial legacy
Source: BMC Evol Biol. 2008 Jan 28;8:26. doi: 10.1186/1471-2148-8-26 (PMC2266909; doi:10.1186/1471-2148-8-26)

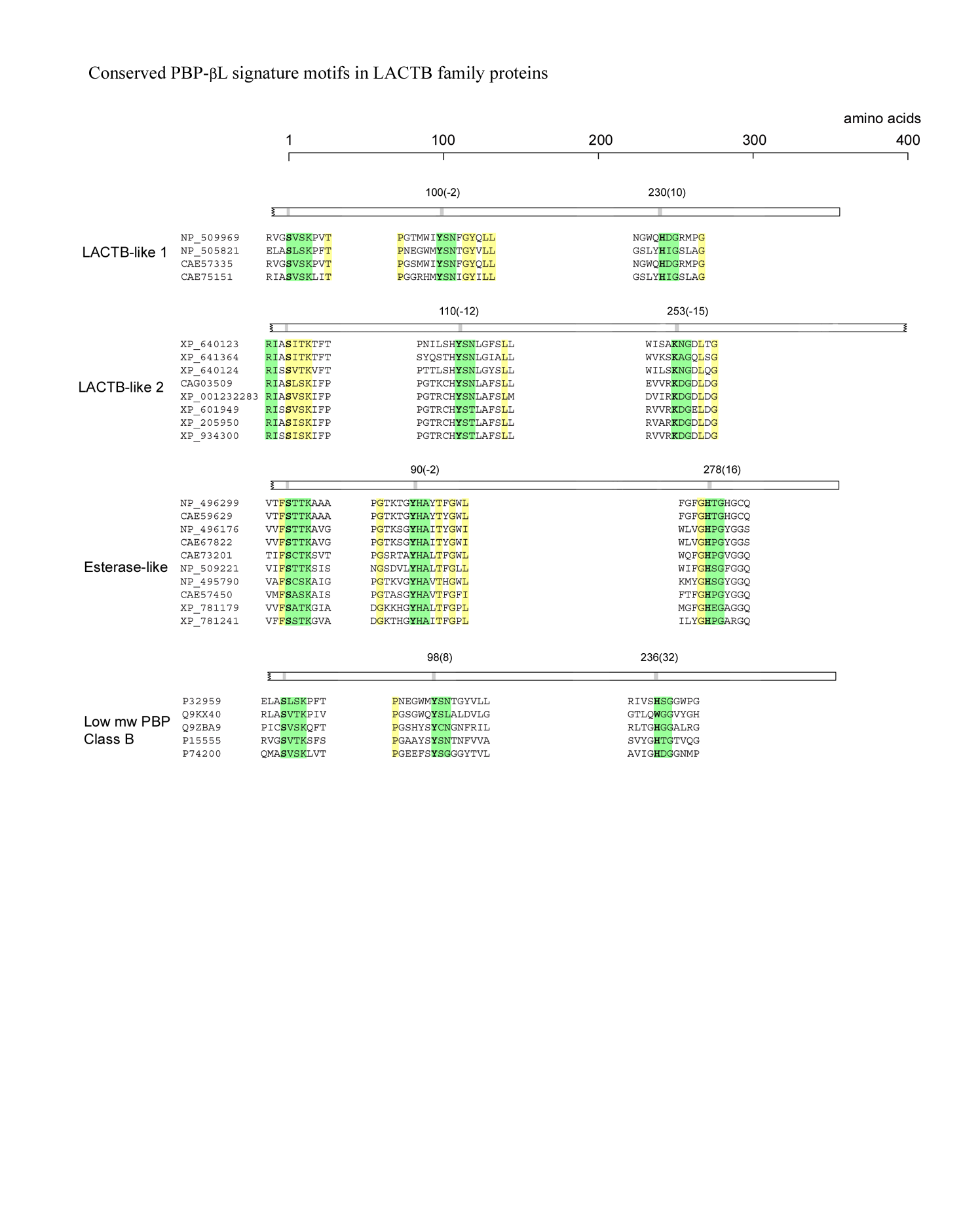

Supplement: Additional file 3 — Conserved PBP-βL signature motifs in LACTB family proteins. Multiple amino acid alignment of PBP-βL signature motif-containing segments from LACTB family proteins. [file 1471-2148-8-26-S3.tiff]
